# Supplementary material for: Prognosis Risk Model Based on Necroptosis-Related Signature for Bladder Cancer
Source: Genes (Basel). 2022 Nov 15;13(11):2120. doi: 10.3390/genes13112120 (PMC9690141; doi:10.3390/genes13112120)
Supplement: Supplementary file 1 [file genes-13-02120-s001.zip › caption for supplementary figures and tables.pdf]

Figure S1. stromal pathway of NEC clusters in meta-BLCA cohort.

Figure S2. NEC score in different Molecular subtypes in meta-BLCA cohort.

Figure S3. Differently expressed NEC score in NEC clusters in TCGA, GSE13507, and GSE32548 cohort.

Figure S4. Kaplan-Meier survival curves of high/low NEC score samples in TCGA, GSE13507, and GSE32548 cohort.

Figure S5. Correlation between NEC score and immune cell infiltration in TCGA cohort.

Table S1. Necroptosis related genes used in our study.

Table S2. The primer sequences of Key gene in NEC signature.

Table S3. Expression matrix of 159 DEGs associated with prognosis.

Table S4. The coefficients of 14 genes establishing the NEC score.

Table S5. The clinical characteristic of the patients included in qPCR.
