# Supplementary material for: Prognosis Risk Model Based on Necroptosis-Related Signature for Bladder Cancer
Source: Genes (Basel). 2022 Nov 15;13(11):2120. doi: 10.3390/genes13112120 (PMC9690141; doi:10.3390/genes13112120)
Supplement: Supplementary file 1 [file genes-13-02120-s001.zip › Table S5.pdf]

Table S5. The clinical characteristic of the patients included in qPCR.

Characteristic of patients		
Gender		
	Male	49
	Female	6
Age		69.8±10.7
T		
	Ta	5
	T1	9
	T2	15
	T3	22
	T4	4
N		
	N0	40
	N+	15
Grade		
	High Grade	47
	Low Grade	8
